# Multi-Leu PACE4 Inhibitor Retention within Cells Is PACE4 Dependent and a Prerequisite for Antiproliferative Activity

**DOI:** 10.1155/2015/824014

**Published:** 2015-05-31

**Authors:** Frédéric Couture, Kévin Ly, Christine Levesque, Anna Kwiatkowska, Samia Ait-Mohand, Roxane Desjardins, Brigitte Guérin, Robert Day

**Affiliations:** ^1^Institut de Pharmacologie de Sherbrooke, Université de Sherbrooke, Sherbrooke, QC, Canada J1H 5N4; ^2^Department of Surgery/Urology Division, Université de Sherbrooke, Sherbrooke, QC, Canada J1H 5N4; ^3^Department of Nuclear Medicine and Radiobiology, Centre d'Imagerie Moléculaire de Sherbrooke, Centre de Recherche du Centre Hospitalier Universitaire de l'Université de Sherbrooke, Sherbrooke, QC, Canada J1H 5N4

## Abstract

The overexpression as well as the critical implication of the proprotein convertase PACE4 in prostate cancer progression has been previously reported and supported the development of peptide inhibitors. The multi-Leu peptide, a PACE4-specific inhibitor, was further generated and its capability to be uptaken by tumor xenograft was demonstrated with regard to its PACE4 expression status. To investigate whether the uptake of this inhibitor was directly dependent of PACE4 levels, uptake and efflux from cancer cells were evaluated and correlations were established with PACE4 contents on both wild type and PACE4-knockdown cell lines. PACE4-knockdown associated growth deficiencies were established on the knockdown HepG2, Huh7, and HT1080 cells as well as the antiproliferative effects of the multi-Leu peptide supporting the growth capabilities of PACE4 in cancer cells.

## 1. Introduction

The development of novel therapeutics for prostate cancer persists as an essential goal to circumvent the resistance of cancer cells to antiandrogen based therapies, which inevitably leads to tumor relapse and life-threatening metastases [1]. Prostate cancer is the second leading cause of cancer death in North American men with the highest prevalence among cancers [2]. Attempts at defining androgen alternative convergence pathways notably congregated to the cancer cell growth factor axis as a replacement supply in growth signaling [3, 4].

Coherent with this path, we have recently identified and validated the proprotein convertase (PC) PACE4 as a novel target for prostate cancer. This serine protease, together with six other members of the PC family, which includes furin, PC5/6, PC7, PC1/3, PC2, and PC4, is responsible for the proteolytic processing of numerous proproteins requiring a cleavage at dibasic site, consensually R-X-(K/R)-R↓ [5, 6]. In prostate adenocarcinomas, PACE4 expression levels are increased and correlate with tumor staging [7, 8]. When its expression is downregulated in prostate cancer cell lines, either using ribozyme [7] or short-hairpin RNAs (shRNA) [9], PACE4-knockdown cells display reduced proliferation rates and clonogenic potential. Moreover, these cells have diminished capabilities in conditioning media with proliferative factors. When further xenografted on athymic nude mice, PACE4-knockdown cells implant as tumors had significantly reduced growth rate and displayed deficient cell-cycle progression capabilities [7, 9]. Similar conclusions were drawn from the ovarian cancer cell model SKOV3 [10]; however, PACE4-specific substrates responsible for the observed phenotypes remain unknown.

These observations prompted us to develop peptide-based PACE4 inhibitors and resulted in the discovery of the leading peptide scaffold, namely, the multi-Leu (ML) peptide (*Ac*-LLLLRVKR-NH_2_), which is a selective PACE4 inhibitor with a 20-fold selectivity for PACE4 over furin, the ubiquitously expressed canonical representative member of the PC family. This compound also displayed antiproliferative properties upon prostate cancer cells [11]. Additionally, we have examined the potential of PACE4 for molecular imaging of prostate cancer using positron-emission tomography (PET) and a ML peptide conjugated to a NOTA (1,4,7-triazacyclononane-1,4,7-triacetic acid) chelating group [12] for radiometal labeling with copper-64 (^64^Cu). Interestingly, very fast peptide uptake associated with PACE4 levels was distinguishable in prostate cancer xenografts and tissues when tumor-bearing mice were injected with ^64^Cu/NOTA-ML, which raised the interrogation of whether PACE4 was capable of retaining the ML peptide by cancer cells. To address this premise, we sought to validate this possibility using prostate cancer cells as well as hepatocellular carcinoma and fibrosarcoma cell models and their respective PACE4-knockdown (shPACE4) counterparts. Using proliferation assays, we showed that these cell models are readily affected by PACE4 downregulation and display a broad variety of PACE4 expression levels in order to evaluate peptide uptake as well as its retention. Our data indicate that ^64^Cu/NOTA-ML peptide uptake and retention within the cell readily require PACE4 but also show that the peptide retention over 2 h postpulse ending correlates with PACE4 levels in these cells. Additionally, we showed that the ML peptide could affect the growth of these cells when compared to an inactive scramble peptide.

## 2. Material and Methods

### 2.1. Cell Culture and Knockdown Cell Lines

All cell lines were obtained from the American Type Culture Collection (ATCC, Mannasas, USA). LNCaP, DU145, and PC3 were cultured in RPMI 1640 supplemented with either 5% (DU145) or 10% (LNCaP) fetal bovine serum (Wisent Bioproducts, St Bruno, QC). HepG2 and HT1080 cells were cultured in EMEM and Huh7 in DMEM plus 10% FBS. For stable PACE4-knockdown cell lines generation, the method described in D'Anjou et al. [13] was used with PACE4 mRNA targeting shRNA (CCTGGAAGATTACTACCATTT, TRCN0000075250; Sigma Aldrich) and cells were further cultured with their selection-requiring puromycin of concentrations 2 *μ*g/mL for HepG2, DU145, and Huh7, 1 *μ*g/mL for LNCaP, and 10 *μ*g/mL for HT1080. All cells were grown at 37°C in a water-saturated atmosphere with 5% CO_2_. Cells were grown in complete media and harvested at their exponential growing state.

### 2.2. Quantitative PCR

Cell total RNA was extracted using Qiagen RNA Isolation Kit (Qiagen, Valentia, CA). Real-time quantitative polymerase chain reaction (qPCR) was performed using the protocol and the primers as previously described [9]. Experiments were repeated on three independent RNA preparations (*n* = 3).

### 2.3. Cell Proliferation Assays

Cell growth rates were compared by seeding 2000 cells in 96-well plates in triplicate. Every 24 h following cell seeding, 2,3-bis-(2-methoxy-4-nitro-5-sulfophenyl)-2H-tetrazolium-5-carboxanilide salt (XTT) reagent (Roche Applied Science, Indianapolis, IN) was added to each well and followed with absorbance read after 4 h according to the manufacturer's instructions. For each time point, data were reported as percentage of mean values measured at 24 hours with corrections applied for the respective blanks (complete medium without cells).

To evaluate peptide growth inhibitory properties, 3000 (Huh7) and 4000 (HepG2) cells were seeded in triplicate in 96-well plates. After 24 h, media was changed for serum-free medium and increasing concentrations of peptide were added. After 72 h, 25 *μ*L of 3-(4,5-Dimethylthiazol-2-yl)-2,5-diphenyltetrazolium bromide (MTT) reagent (5 mg/mL; Sigma Aldrich) was added to each well and, after 4-hour incubation, medium was carefully removed and cells were lysed in acidic isopropanol (24 : 1 HCl 1 N). Absorbance at 550 nm and 650 nm (reference wavelength) was measured using a plate spectrophotometer.

### 2.4. Peptide Radiolabeling

Labeling of a NOTA-ML was performed as reported previously [12] using ^64^Cu prepared via the ^64^Ni(p,n)^64^Cu reaction using an enriched ^64^Ni target electroplated on a rhodium disk [14]. The peptide was labeled with ^64^Cu under previously optimized conditions [15] as follows: a peptide (5 *μ*g) was incubated with [^64^Cu]Cu-(OAc)_2_ (300−370 MBq; 8−10 mCi) in ammonium acetate buffer (0.1 M, pH 5.5) and heated at 95°C for 10 min. Next, the labeled peptide was applied on a C18 Sep-Pack column (Waters, Milford, MA) to eliminate trace of free ^64^Cu with water washes and further eluted using ACN containing 0.025% TFA. ACN was evaporated and peptide preparation was counted in a Capintec radioisotope calibrator (Capintec, Inc., NJ, USA) to calculate the specific activity of the product. The resulting ^64^Cu peptide was reconstituted in PBS at pH 7.4. Full peptide labeling was routinely assessed by HPLC.

### 2.5. Cell Uptake

For cell uptake experiments, 2.5 × 10^5^ cells were seeded into 12-well plates 48 h before the experiments were performed. Prior to peptide addition, culture media was replaced by fresh serum-free medium and 180–200 KBq (5 *μ*Ci; 30 *μ*L) of ^64^Cu-NOTA-ML was added to each well. After each incubation time, cells were washed three times with PBS and lysed using 10% SDS. Cell lysate radioactive content was measured in a gamma counter (Cobra II autogamma counter, Packard, MN). For efflux studies, cells were allowed to uptake radiolabeled peptide for 2 h before being washed with PBS and efflux with fresh medium for the indicated incubation times was allowed. Precise cell counts (Trypan blue stain) were determined from similar plates following the same washing procedure to avoid cell detachment bias. The results were expressed as percentage of the peptide dose per 10^6^ cells.

### 2.6. Peptide Synthesis

All compounds used in this study were prepared as previously described [12, 16].

## 3. Results

**Figure 1:**
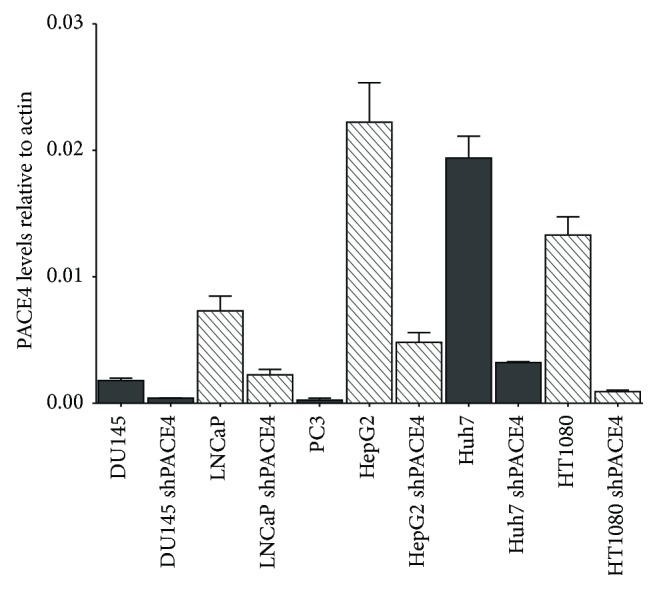
PACE4 expression levels in the studied cell lines. Quantitative PCR (qPCR) measurement of PACE4 expression levels in both control and PACE4-knockdown cell lines. Data are means ± SEM of mRNA levels from at least 3 independent cell cultures using *β*-actin as the reference housekeeping gene.

To address the relation between ^64^Cu/NOTA-ML cell uptake and PACE4 status, an array of cell lines was screened for their endogenous expression levels of PACE4 by qPCR. Among prostate cancer cells, LNCaP cells have previously been reported to have higher levels of PACE4 than DU145 cells, whereas PC3 cells are PACE4-negative [11]. To further increase the range of PACE4 expressing cells, HepG2 and Huh7 hepatocellular carcinoma cell lines as well as HT1080 fibrosarcoma cells were assayed for their relative PACE4 mRNA levels (Figure 1). All these cells expressed PACE4 at variable levels, for example, HepG2 having the highest levels, directly followed by Huh7 and finally by HT1080, which had levels close to LNCaP cells. HepG2 cells were previously reported to express considerably high PACE4 levels [17], just like liver cells [18] and HT1080 were also known to express PACE4 [19]. To provide an appropriate control for each of these cell lines, a stable PACE4-knockdown cell line was generated for each of these PACE4-expressing cell lines using lentiviral transduction of PACE4-targeting shRNA. In each case, stable transduction yielded at least a 70% reduction of PACE4 expression in knockdown cells when compared to its respective controls (Figure 1), thus providing an even larger array of PACE4 expression levels.

**Figure 2:**
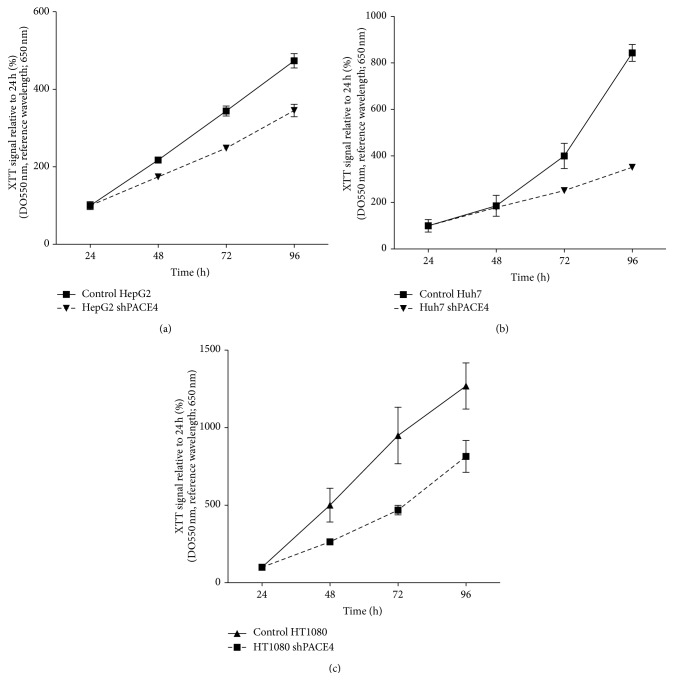
Knockdown cell proliferation rates. Percentage of initial (24 h postseeding) metabolic activity determined on mirror plates every 24 h using XTT reagent for HepG2 (a), Huh7 (b), and HT1080 (c). Control cells are shown as plain line and knockdown cells as dashed lines. Data are means ± SEM of at least 3 independent experiments.

It was previously demonstrated that PACE4 knockdown in DU145 and LNCaP prostate cancer cells was associated with markedly reduced cell growth rate both* in vitro* and* in vivo* [9]. Since PACE4 knockdowns in HepG2, Huh7, and HT1080 were never reported before, we sought to evaluate whether this growth phenotype was also observable. XTT proliferation assays were carried out to measure the proliferation rates of these cells compared to their respective control. As shown in Figure 2, cell growth was monitored on 96 h and shPACE4 reduced cell proliferation by 30% for HepG2, 45% for Huh7, and 35% for HT1080 after 96 h relative to the control cells. These growth rate reductions were however lower than those observed in the prostate cancer cells (i.e., reduction of about 50% of growth after 96 h) [9], indicating a relevant but yet inferior PACE4 dependance on their growth capabilities.

**Figure 3:**
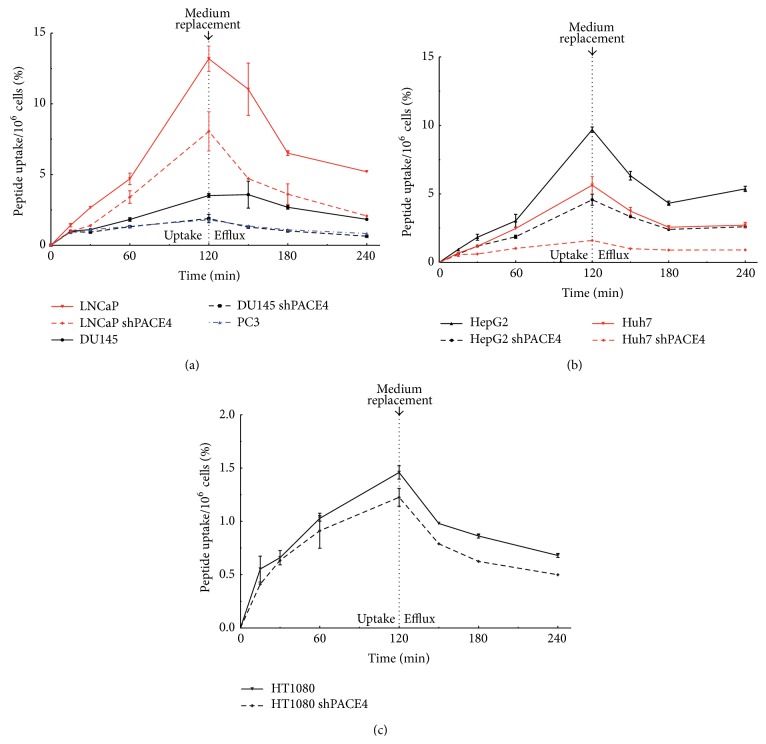
^64^Cu/NOTA-ML uptake and retention kinetics in cells. Percentage of the initially applied radioactivity in prostate cancer (a), hepatocellular (b), or fibrosarcoma (c) cell lines after different pulse times. Cells were lysate following two extensive PBS washes. For retention, a 2-hour pulse was performed followed by washes and different efflux time points. Control cells are shown as plain line and knockdown cells as dashed lines. Data are means ± SEM of at least 3 independent experiments.

**Figure 4:**
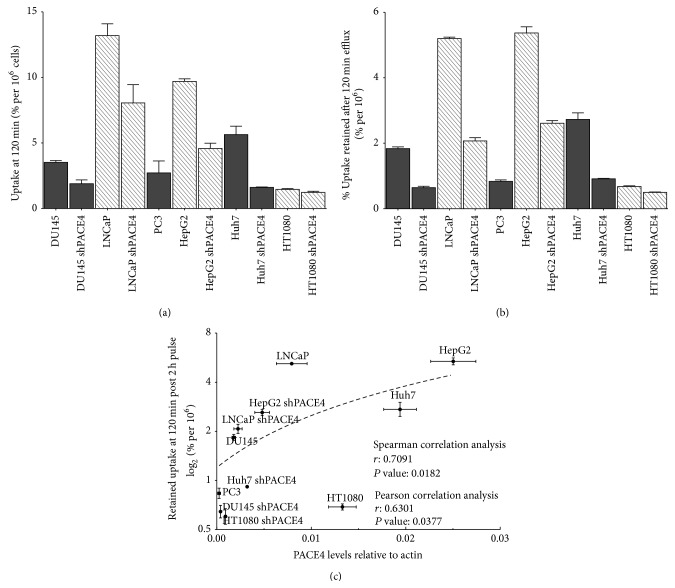
^64^Cu/NOTA-ML uptake and retention correlation with PACE4 levels in cells. (a) Percentage of uptake after 2-hour pulse and (b) percentage of retained radioactivity 2 h after the 2-hour pulse per 1 × 10^6^ cells. (c) Plot of PACE4 expression levels as a function of the log_2_ of the mean percentage of retained radioactivity after 2-hour efflux. Log_2_ is applied only to scatter points to make it more visible. Name of the cell line is indicated above each corresponding point. Dashed line represents a linear regression for visual purposes.

To assess peptide uptake in these cells, ^64^Cu/NOTA-ML was prepared with high specific activity (1900–2000 Ci/mmol). Labeled peptide uptake on these cell lines was evaluated by the addition of 5*μ*Ci (around 2.5 pmol or 2 ng) to their culture medium. Cell uptake was further assessed during a 2-hour pulse by calculating both peptide at the surface and incorporation in the cells after medium removal and extensive cell washes with PBS. Following a 2-hour pulse, cells were also thoroughly washed from their peptide-containing medium and efflux of the unbound peptide fraction up to an additional 2 h was allowed in order to calculate compound retention to the cells (Figure 3). Interestingly, the ^64^Cu/NOTA-ML uptakes are significantly reduced in all PACE4-knockdown cells tested when compared to the uptakes in their respective control cells. In prostate cancer cells (Figure 3(a)), PACE4-knockdown diminished ^64^Cu/NOTA-ML uptake by 40% in both LNCaP (8 versus 13%/10^6^ cells after 2 h) and DU145 cells (1.8 versus 3.6%/10^6^ cells after 2 h). Interestingly, ^64^Cu/NOTA-ML uptakes in DU145 shPACE4 were comparable to the very low peptide uptake observed in the PACE4-deficient PC3 cells (1.8%/10^6^ cells after 2 h). In HepG2 cell models, uptake reached ^64^Cu/NOTA-ML 9.6%/10^6^ cells compared to 4.6% in the control and shPACE4 cells, respectively, yielding a disparity greater than 50% (Figure 3(b)). Interestingly, in the Huh7 cells this difference was even bigger (5.5 versus 1.6%/10^6^ cells) representing more than 70% uptake inhibition. Surprisingly, the radiolabeled peptide had a very low uptake in HT1080 cells (1.46%/10^6^ cells); however, it was still higher than the one of its PACE4-knockdown counterparts (1.22%/10^6^ cells; Figure 3(c)). Since peptide radiotracer degradation is negligible during the 2-hour timeframe of this experiment [16] and due to the tight and stable ^64^Cu chelation in the NOTA moiety [20], the possibility of any artifacts associated with these parameters can be excluded. These differences can be clearly visualized when directly comparing the percentage of uptake after 2 h (Figure 4(a)) and even more clearly when comparing the percentage of ^64^Cu/NOTA-ML retained after a 2-hour efflux (Figure 4(b)) encompassing the fact that PACE4 levels are readily reflecting the ML peptide uptake. When percentage of a radiolabeled peptide retained in the cells 2 h postpulse was plotted together with PACE4 expression levels (Figure 4(c)), a positive and significant correlation was observed (Spearman *r* coefficient: 0.7091, *P* value: 0.0182), which was not the case with neither the percentages of uptake after 2 h (*P* value: 0.1457, data not shown) nor furin expression levels (*P* values: 0.1815 and 0.3578 for uptake and retention values resp.). These data indicate that a major part of the ^64^Cu/NOTA-ML retention directly correlates with PACE4 levels but not with cell entry or furin levels.

**Figure 5:**
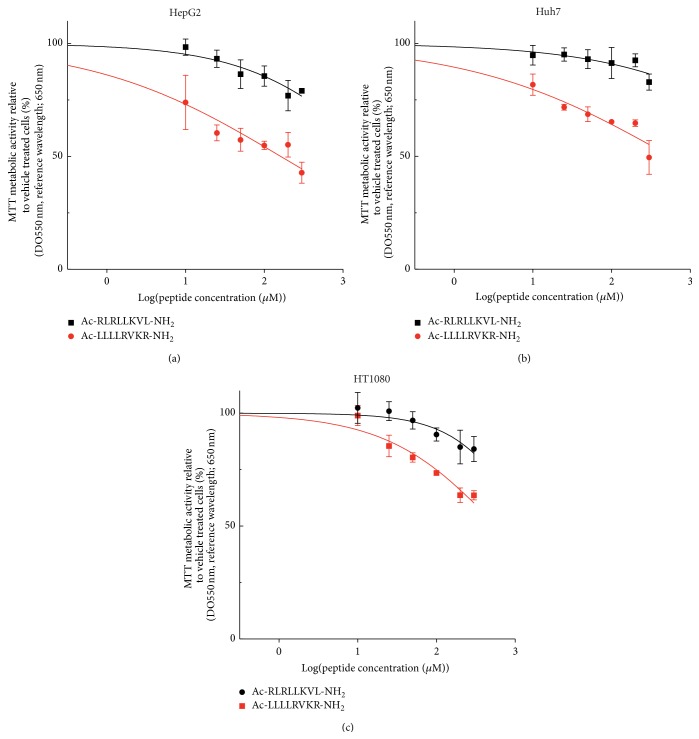
Antiproliferative properties of ML peptide on the tested cell lines.* Ac*-ML-NH_2_ and its scramble version were applied with various concentrations ranging from 1 *μ*M to 300 *μ*M to assess dose-response inhibition of cell proliferation relative to untreated cells on (a) HepG2, (b) Huh7, and (c) HT1080. Data are means ± SEM of at least 3 independent experiments.

Based on PACE4-associated growth capabilities denoted by the XTT proliferation assays (Figure 2), growth inhibitory properties of the ML peptide were assayed on these new, yet uncharacterized cell models to evaluate pharmacological response to PACE4 inhibitor knowing their cell penetration properties. Therefore, increasing concentrations of the ML peptide (*Ac*-LLLLRVKR-NH_2_) were applied on HepG2, Huh7, and HT1080 cells to assess proliferation inhibition compared to untreated cells. The scramble version of the ML peptide (*Ac*-RLRLLKVL-NH_2_) was used as a negative control since it has the same amino acid composition and presents very little* in vitro* inhibition (*K*_*i*_: >10 *μ*M) [16] properties against recombinant PACE4 likely due to impaired dibasic consensus motif compared to* Ac*-ML-NH_2_ (*K*_*i*_: 20 nM) independently of the addition of the NOTA moiety [12]. As shown in Figure 5, the ML peptide yielded a dose-response inhibition of cell proliferation when compared to the scramble inactive peptide.

## 4. Discussion

In prostate cancer as well as in numerous types of cancer, attempts at defining novel or alternative “druggable” pathway to block tumor progression converged toward growth factors signaling pathways, for example, invasion, lymphangiogenesis, and angiogenesis [21, 22]. Since growth factors homeostasis strongly depends on proteolysis for either their release [23] or their full activation [22], the targeting of protease has now become an important field in cancer research [24]. This is notably highlighted in mouse transgenic models where cancer progression was concomitant with the increasing activation of matrix metalloproteinases [25], which are known for their roles in growth factors thus increasing their bioavailability [26].

PACE4, along with some other PCs [22, 27–30], has previously been demonstrated to be a relevant target for cancer therapeutic development [7]. Moreover, our research group has recently generated the first PACE4-specific peptide inhibitor in an effort to pharmacologically alter PACE4 activity in tumor cell. Knowing that the broadly expressed PACE4 is overexpressed in prostate cancer cells, we previously evaluated the possibility that higher PACE4 levels in cancer cells could yield tumor specific accumulation of the peptide and in turn could benefit both therapeutic and tumor detection point of views. ^64^Cu/NOTA-ML* in vivo* distribution has been examined and tissues accumulation [12] matched, at least in part, with known PACE4 expression levels in organs [18]. However, validation of PACE4-specific uptake required more attention to understand both the capabilities of the peptide to determine PACE4 status in tumor and its target reaching in therapeutic interventions.

On the other hand, it has previously been shown that cell penetration properties of the ML inhibitor were an important factor to mediate its full growth inhibition effects, as depicted by the addition of a polar hydrophilic polyethylene glycol (PEG8) moiety, which blocked cell entry and its antiproliferative effects [11]. Fluorescein-labeled peptide also showed diffuse pattern within the cell once checked by confocal analyses. This diffuse entry pattern was associated with the high hydrophobic potential of the tetra-leucine core. This latter phenomenon may explain the fact that ^64^Cu/NOTA-ML uptake, in opposition to retained signal after 2 h (Figure 4(a)), did not correlate with PACE4 expression levels as the presence of high peptide concentration kept intracellular and extracellular compartment into equilibrium until cells were washed but predominantly allowed for efflux of unbound peptide. In fact, this phenomenon brings conditions similar to the one taking place* in vivo* as the peptide clearance from the circulation is extremely fast (within 10–15 min) allowing tumors and tissues to efflux part of the uptaken peptide [12] while maintaining stable levels within the tumor.

The use of various cell types with their corresponding PACE4-knockdown equivalent allowed the depiction of a clear correlation between the peptides retained by the cells and their levels of PACE4 expression even when comparing all cells together (Figure 4(c)). Surely, numerous parameters differ between these cell types such as their membrane composition and permeability, endocytosis rates, and macropinocytosis rates which may, in part, affect total cell uptake [31, 32]. This further encompasses the importance of using a matched PACE4-knockdown cell line to avoid intercell type variability and decoys when assessing peptide uptake and retention.

As an example, the inhibitory properties were considerably lower than those observed on prostate cancer cells, which is coherent with the PACE4-knockdown growth inhibition shown in Figure 2. The HT1080, which was still considerably affected by PACE4 downregulation (Figure 2(c)), was also showing the lowest ^64^Cu/NOTA-ML uptake among PACE4-expressing cells (Figure 3(c)) and consequently had the lowest response to the ML peptide, which is coherent with the necessity of peptide entry to exert its full growth inhibition properties.

## 5. Conclusion

Our data indicate that the ^64^Cu/NOTA-ML retention within the cell readily requires PACE4 but also show that peptide retention over 2 h postpulse positively correlates with PACE4 levels in these cells. Additionally, we demonstrated that the ML peptide could readily affect the growth of these cells when compared to its inactive scramble version. These results confirm the notion that peptide entry within cell is an important requirement to exert growth inhibition properties as determined by comparing PACE4 silencing to the ML peptide response. This study further encompasses the possibility to use the ML-based techniques to determine PACE4 status in tumors.

## Abbreviations

^64^Cu: Copper-64
ML: Multileucine peptide
MTT: 3-(4,5-Dimethylthiazol-2-yl)-2,5-diphenyltetrazolium bromide
NOTA: 1,4,7-Triazacyclononane-1,4,7-triacetic acid
PC: Proprotein convertase
qPCR: Quantitative polymerase chain reaction
shRNA: Short-hairpin RNA
XTT: 2,3-Bis-(2-methoxy-4-nitro-5-sulfophenyl)-2H-tetrazolium-5-carboxanilide.
